# Signaling metabolite β-aminoisobutyric acid as a metabolic regulator, biomarker, and potential exercise pill

**DOI:** 10.3389/fendo.2023.1192458

**Published:** 2023-05-29

**Authors:** Xuejie Yi, Yang Yang, Tao Li, Menghuan Li, Tingting Yao, Guangxuan Hu, Genmeng Wan, Bo Chang

**Affiliations:** Social Science Research Center, Shenyang Sport University, Shenyang, Liaoning, China

**Keywords:** BAIBA, exercise mimic, biomarker, metabolic regulation, inflammation, ROS

## Abstract

Signaling metabolites can effectively regulate the biological functions of many tissues and organs. β-Aminoisobutyric acid (BAIBA), a product of valine and thymine catabolism in skeletal muscle, has been reported to participate in the regulation of lipid, glucose, and bone metabolism, as well as in inflammation and oxidative stress. BAIBA is produced during exercise and is involved in the exercise response. No side effect has been observed in human and rat studies, suggesting that BAIBA can be developed as a pill that confers the benefits of exercise to subjects who, for some reason, are unable to do so. Further, BAIBA has been confirmed to participate in the diagnosis and prevention of diseases as an important biological marker of disease. The current review aimed to discuss the roles of BAIBA in multiple physiological processes and the possible pathways of its action, and assess the progress toward the development of BAIBA as an exercise mimic and biomarker with relevance to multiple disease states, in order to provide new ideas and strategies for basic research and disease prevention in related fields.

## 1 β-aminoisobutyric acid: a signaling metabolite produced in response to exercise

Organisms maintain their life activities through the transformation of substances into energy; small-molecule products in this process are called metabolites (1). Some metabolites that participate in the transmission of information between cells are referred to as signaling metabolites (2). They represent important control points in homeostasis and may serve as biomarkers of diseases. Previous studies had suggested β-aminoisobutyric acid (BAIBA) as a muscle factor (myokine); however, BAIBA, being an amino acid metabolite, is neither a protein nor an active peptide, and may be more appropriately referred to as a muscle signaling metabolite.

BAIBA is a catabolic metabolite of thymine and valine in skeletal muscle (3). It is present as D- and L-enantiomers in mammals, D-BAIBA being an intermediate product of thymine degradation in the cytoplasm, and L-BAIBA being produced by L-valine decomposition (3). In 2014, Roberts et al. (4) had observed a significant increase in BAIBA synthesis in myoblasts, after PGC-1α treatment, by liquid chromatography-liquid mass spectrometry (LC-MS). Subsequent *in-vivo* and *ex-vivo* experiments confirmed that BAIBA might promote the differentiation of pre-adipocytes into brown fat, promoting free fatty acid (FFA) oxidation (4, 5) Further, BAIBA was considered to indirectly up-regulate browning and fatty acid oxidation in the liver, maintain lipid homeostasis, and prevent and improve lipid metabolism disorders (4, 6–15). In addition, BAIBA was reported to improve glucose homeostasis in mice (8, 13, 16); it could regulate the differentiation of osteoprogenitor cells, affect the balance between the number of osteoblasts and osteoclasts, maintain bone mass homeostasis (17, 18), reduce inflammatory response and oxidative stress (17, 19), and improve related diseases (4, 8, 14, 15, 20). Overall, BAIBA has drawn abundant attention of researchers recently.

The synthesis and metabolism of BAIBA are closely related to exercise, and no adverse effect has been observed in subjects, animals, or cells receiving different doses of BAIBA, suggesting its potential to be developed as an exercise pill. In addition, BAIBA has been associated with the development of osteoporosis and neo-coronary pneumonia; however, differences in conditions used by different investigators may be fundamental to BAIBA’s role as a disease marker. The only review on BAIBA till date has reported its relationship with lipid metabolism (21), and the mechanisms of action of BAIBA are still mostly speculative. Therefore, in this review, we discussed the biological functions of BAIBA, its relationship with exercise, drug applicability, and considerations of it being a disease marker, in order to provide a theoretical basis for the study of BAIBA and related diseases.

## 2 BAIBA metabolism process

Thymine and dihydro pyrimidine dehydrogenase ((DPYD)) and hydrogen reacted to form two Thymine (Dihydrothymine) (22). Dihydrothymine combined with dihydropyrimidinase (DPYS) to form N-carbamoyl-β-amino-isobutyric acid (N-carbamoyl-BAIBA). After further reaction of D-BAIBA by N-carbamoyl-BAIBA and beta-ureidopropionase (UPB1), D-methylmalonic semialdehyde (D-MMS) was finally produced through AGXT2 in mitochondria (23). Studies have detected the expression level of AGXT in various organs such as the brain, liver, pancreas, spleen, heart, lung, esophagus, stomach, small intestine, colon, skeletal muscle, kidney, thymus, testis, ovary, uterus, prostate, tongue and skin. It was found that the expression level was higher in the liver and kidney.

L-BAIBA was produced by catabolism of the branched amino acid L-VALINE (24). L-VALINE was formed under ammonia, and the oxidation reaction of methyl malonyl half aldehyde (L-methylmalonylsemialdehyde, L-MMS). L-MMS produced L-BAIBA in reaction with mitochondrial enzyme 4-aminobutyrate aminotransferase (ABAT) (25, 26). It has been reported that the production of L-BAIBA by ABAT is a bidirectional reaction, so the same enzyme can catalyze the conversion of L-BAIBA to L-MMS. At the same time, the Malonate – semialdehyde dehydrogenase (MMSDH), can L-oxide MMS and D-MMS propionyl coa (24). L-BAIBA is converted to D-BAIBA and vice versa through the stereoisomerization pathway between L-MMS and D-MMS (24, 27). MMSDH has been less reported in organs and seems to be concentrated in the liver.

## 3 Biological functions of BAIBA

### 3.1 BAIBA and lipid metabolism

#### 3.1.1 BAIBA promotes browning of white fat and enhances fat metabolism

Fat is an important energy source that provides energy to the body in the presence of a variety of enzymes and is important for maintaining life activities. However, disturbances in fat metabolism can induce the development of diseases, such as obesity and non-alcoholic fatty liver (28). The 2022 a study reported no significant change in body weight and fat mass in mice fed a high-fat diet along with BAIBA intervention, compared to that in the normal diet group (20), suggesting that BAIBA might play an important role in the prevention of high-fat diet-induced fat deposition. Fat tissue can be classified morphologically and functionally as white, brown, or beige. White fat cells have large lipid droplets and few mitochondria, which facilitate energy storage. Brown fat cells have small lipid droplets and many mitochondria, which facilitate the oxidative release of energy. Beige fat cells have properties intermediate between those of white and brown fat cells. When stimulated by cold or exercise, white fat cells can convert to beige or brown fat cells (29). Both beige and brown fat present highly specific molecular markers, such as the beige marker T box 1 transcription factor (TBX1), the browning marker uncoupling protein 1 (UCP1), cell death-inducing DFFA-like Effector A (CIDEA), PR domain-containing 16 (PRDM16), and elongase of very long chain fatty acids (ELOVL3) (30).

BAIBA treatment induced morphological changes and increased the levels of browning markers in pre-adipocytes (5). Comparing the effects of different doses of BAIBA and differentiation time on morphological changes and beige/browning markers of lipid droplets in 3T3-L1 (preadipocytes) cells, the number of lipid droplets were found to be increased and the surface area decreased in 3T3-L1 cells, after high BAIBA intervention, on day 4 of differentiation. Beige markers TBX1, browning markers UCP1, CIDEA, PRDM16, and ELOVL3, and the mitochondrial biogenesis marker mRNA were significantly increased, although the effects disappeared after 10 days, suggesting that BAIBA acts early in differentiation. Previous studies had shown that PGC-1α, a functional factor in white fat browning, alleviates mitochondrial dysfunction and increases FFA oxidation in obesity (4, 31). PGC-1α treatment of myocytes led to an increase in BAIBA synthesis, along with increased FFA oxidation, and up-regulation of PPARα, a transcriptional activator. BAIBA enhanced the expression of several other markers in pluripotent stem cells via PPARα (4). This effect was dose-dependent, suggesting that BAIBA could improve lipid deposition by increasing white fat browning, up-regulating coupling, inhibiting phosphorylation, down-regulating adenosine triphosphate (ATP) production, converting bioenergy to heat, and promoting FFA metabolism (4). Overall, it improved lipid deposition.

In summary, BAIBA may regulate lipid metabolism by promoting FFA oxidation through PPARα-mediated white fat browning, and this regulation may occur early in adipocyte differentiation (4, 5, 20). Studies on BAIBA-mediated changes in lipid metabolism are still scarce, and the possible involvement of BAIBA in white fat browning under cold stimulation or fasting is yet be explored.

#### 3.1.2 BAIBA regulates hepatic lipid metabolism

BAIBA may affect fat metabolism in the liver, the main site for FFA synthesis and oxidation. *In-vivo* studies have shown that BAIBA promotes FFA oxidation in the livers of normal mice (4, 6), whereas *in-vitro* experiments suggested no significant change in FFA oxidation following BAIBA treatment of hepatocytes (10). Liver lipid browning is mainly regulated by sympathetic activity, and L-BAIBA is known to improve oxidative stress in H_2_O_2_-treated mouse pheochromocytoma cells (32). BAIBA may not directly regulate hepatic FFA oxidation but might play an indirect role (4, 6, 10, 32, 33); confirmation will require further exploration of nerve-liver crosstalk to clarify the specific regulatory role of BAIBA.

In FFA synthesis, phosphorylated adenosine 5’-monophosphate-activated protein kinase (AMPK) inhibits hepatic fatty acid and triglyceride (TG) production and downregulates FFA synthase expression by inactivating sterol regulatory element-binding proteins (SREBPs)-1c through autophosphorylation. Shi et al. (13) reported that BAIBA attenuated glucosamine-induced ER stress in human hepatocellular carcinoma HepG2 cells and ameliorated the decrease in AMPK phosphorylation under pathological conditions, suggesting that activation of AMPK by BAIBA during ER stress might facilitate lipid metabolism and reduce intracellular lipid ectopic accumulation in hepatocytes (13). Interestingly, the study reported no significant change in ER stress in the medium without the addition of aminoglucose. The latest study in this regard has shown that aminoglucose ions and glucose play a central role in lipid metabolism by synergistically regulating SREBPs expressed in HepG2 and ER membranes, which are activated by ER-Golgi-nuclear translocation processes, suggesting that aminoglucoseions may mediate BAIBA regulation of lipid metabolism. The results suggested that ammonia ions may be critical in mediating the regulation of hepatic lipid synthesis by BAIBA. However, further studies would be required to prove this inference (34).

Although the role and mechanism of BAIBA in hepatic FFA oxidation and white fat browning are not fully clear yet, ammonium ions may play a role in regulating hepatic lipid synthesis in pathological states, suggesting possible future research directions (4, 8–10, 13, 33, 35).

Prevention of elevated lipid levels in blood could be an important approach for treating abnormal lipid metabolism. The role of BAIBA in lipid homeostasis is currently controversial, with some studies showing over expression of PGC-1α, a factor upstream of BAIBA in skeletal muscle, to not affect plasma lipid levels (35). Similarly, oral administration of BAIBA to lean mice had no significant effect on serum lipid parameters, including FFA, TG, total cholesterol, and phospholipid concentrations (9). Shimba et al. (35) reported no significant change in serum TC, HDL-c, LDL-c, or TG levels in ApoE-knockout mice (ApoE-KO) following oral administration of BAIBA. The findings suggested that BAIBA could be an important factor in the maintenance of lipid homeostasis. Shi et al. (13) observed that oral administration of BAIBA upregulated serum fasting FFA, TG, and LDL levels in mice with high-fat diet/low-dose streptozotocin-induced type 2 diabetes mellitus (T2DM). Considering that blood plays an important role in transporting glycerol and FFA, which are carried to the liver or other organs for synthesis of TG or breakdown by oxidation, BAIBA may upregulate lipolysis in adipocytes to compensate for the decrease in serum FFA levels or even upregulate serum FFA levels and maintain lipid homeostasis.

**Figure 1:**
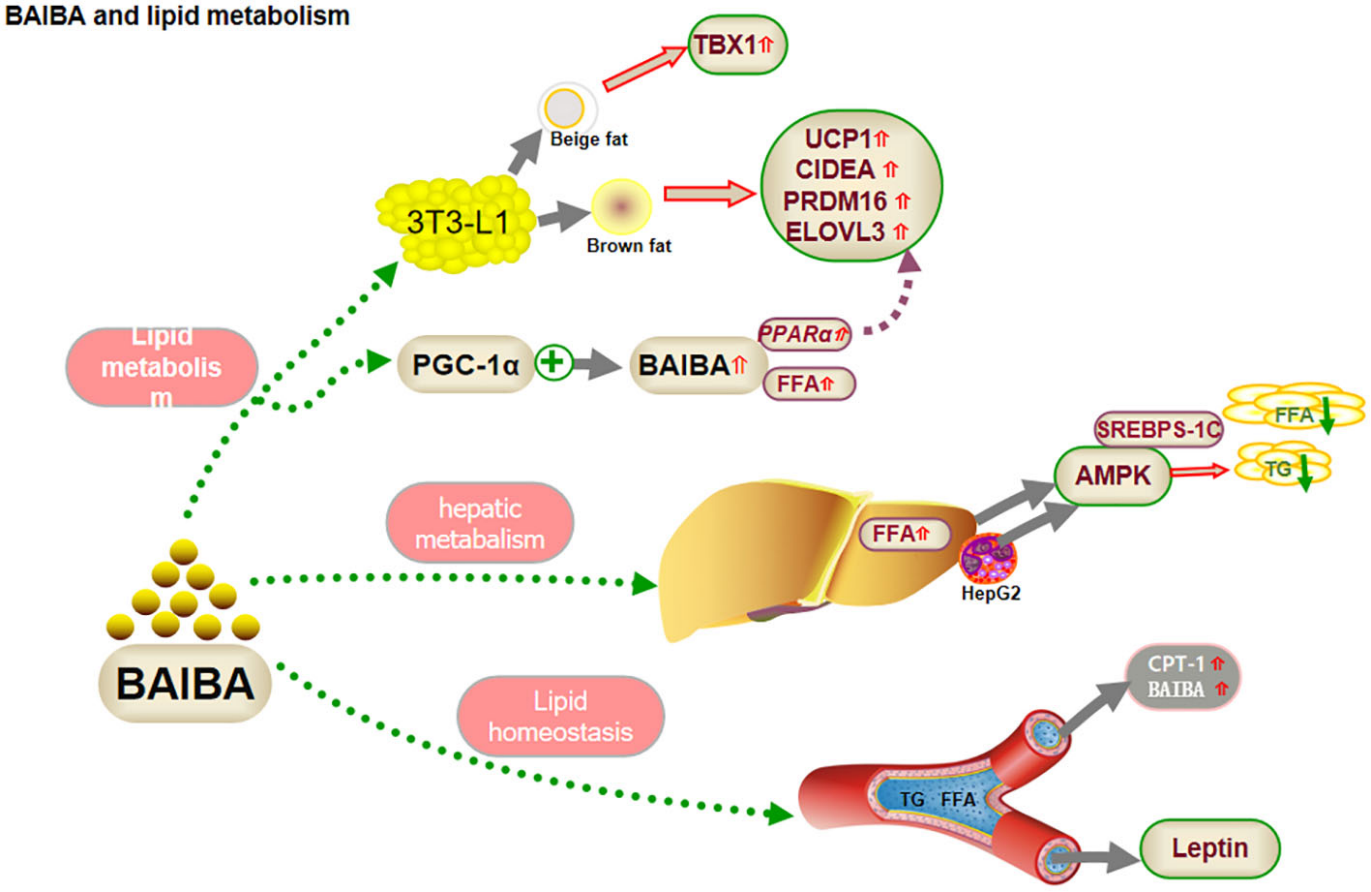
BAIBA and lipid metabolism.

Leptin promotes FFA oxidation in humans and mice (11, 12, 36). Leptin-deficient obese ob/ob mice showed no change in lipid parameters after BAIBA treatment (9, 10), although heterozygous ob/+ mice showed low postprandial TG and fasting cholesterol levels (10). D-BAIBA is degraded by alanine-glyoxalate aminotransferase 2 (AGXT2) to D-methylmalonate semialdehyde (MMS) in mitochondria (23). Rhee et al. (37) showed that serum BAIBA is downregulated after AGXT2 mRNA knock out, suggesting that AGXT2 may regulate lipid metabolism via a response to L-BAIBA or other substrates; future studies would need to monitor the changes in L-BAIBA or other substrates to verify the role of BAIBA in lipid regulation (**Figure 1**).

### 3.2 BAIBA affects carbohydrate metabolism

**Figure 2:**
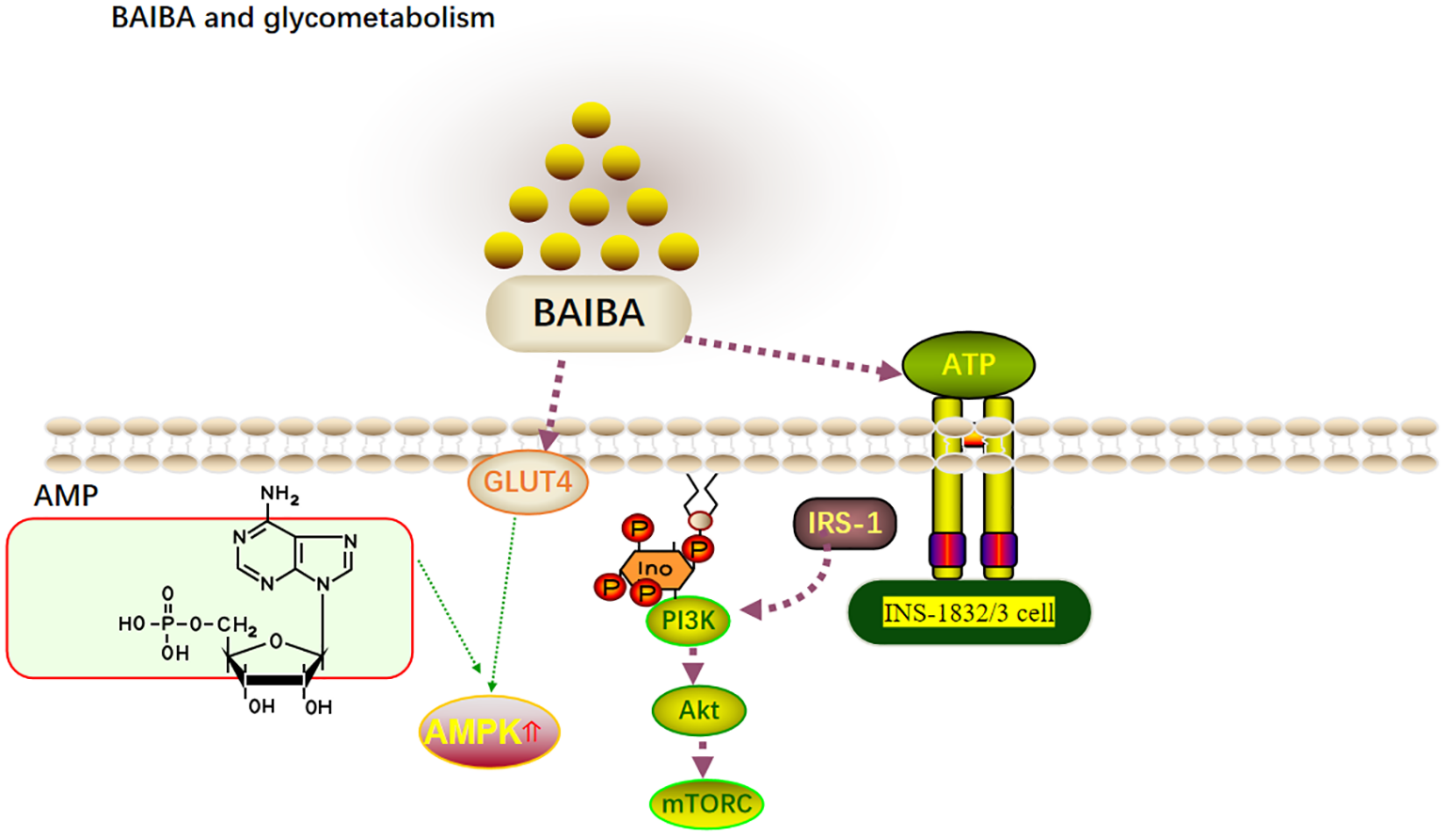
BAIBA and glycometabolism.

BAIBA significantly lowers blood glucose levels in T2DM mice and in mice with insulin resistance (IR). At the same time, one large human cohort study showed serum BAIBA levels to be negatively correlated with serum insulin concentrations (4). BAIBA has been hypothesized to possibly regulate blood glucose by regulating insulin levels; however, since the mentioned study was cross-sectional, the causal relationship between BAIBA and insulin remains unclear. Mitochondria in pancreatic b-cells influence the oxidative phosphorylation of glucose to regulate ATP synthesis and eventually insulin secretion (38). Barlow et al. (39) reported that BAIBA downregulates mitochondrial energy metabolism and insulin release in INS-1832/3 cells *in vitro*. However, this was seen only upon stimulation with the next highest concentration of glucose, and BAIBA had no significant effect on insulin release in INS-1832/3 cells upon glucose and palmitic acid addition. Glucose uptake by tissues can occur through both insulin-dependent and insulin-independent pathways (40), and BAIBA has been speculated to regulate blood glucose levels in IR or T2DM in an insulin-independent manner. However, it has also been shown that glucose uptake by tissues may occur through both insulin-dependent and non-insulin-dependent pathways (40). Insulin is the only hormone in the body that lowers blood glucose levels; however, it also promotes lipid synthesis (41). PI3K-AKT-mTOR is the classical pathway that responds to insulin signaling, with insulin binding to cell surface receptors and activating the PI3K-AKT pathway via IRS1 to directly promote glucose uptake while activating mTORC via AKT to further utilize glucose (42). Increased AMPK activity is positively correlated with GLUT4 translocation to the plasma membrane and rapid glucose uptake (43). Although several experiments with BAIBA intervention (8, 13, 16) had shown serum insulin levels to remain unaltered, significant reductions in blood glucose were observed, and significant improvements in AMPK, AKT, and IRS-1 expression were observed, suggesting that BAIBA may increase insulin sensitivity and lower blood glucose without affecting insulin secretion (13, 16). Future studies on the role of BAIBA in the regulation of glucose metabolism and its mechanisms may represent a new direction for the treatment of IR and metabolic diseases (**Figure 2**).

### 3.3 BAIBA affects bone metabolism

Previous studies had suggested that the action of skeletal muscle on bone is mediated exclusively through mechanical stimuli; recent studies have shown that BAIBA can exert effects on bone proliferation and differentiation (44). Wang et al. (45) reported a positive correlation between hip T-score and serum D-BAIBA levels in healthy elderly, and bioinformatic analysis showed the genes related to bone metabolism to be closely associated with the synthesis and action of BAIBA, suggesting that BAIBA is a potential signaling molecule for bone metabolism. TGF-b promotes early differentiation and matrix production while inhibiting late differentiation and matrix mineralization, and serum BAIBA levels were correlated with TGF-β expression in patients with Duchenne muscular dystrophy when BAIBA was used in an intervention (46); this suggested that BAIBA might regulate bone metabolism via osteoprogenitor cells.

Osteoblasts are multifunctional cells that regulate bone reconstruction (18, 47). Kitase et al. (18) observed that oral administration of L-BAIBA reversed the reduction in bone trabecular volume in mice after two weeks of hindlimb suspension, and that mRNA expression of osteoclast metabolic factors was not affected by suspension. The regulation of bone metabolism by L-BAIBA has been suggested to not occur through sclerostin or RANKL/OPG regulation of osteoblasts. As mentioned above, the improved reduction in trabecular volume and the induction of BMSCs in the bone matrix by TGF-b to migrate to sites of bone resorption to promote osteoblast formation (46, 48–50). This suggested that the regulation of bone metabolism by BAIBA may revolve around osteoblasts rather than osteoclasts.

Reactive oxygen species (ROS) are important messengers in osteoblast proliferation and differentiation, and play an important role in the proliferation of pre-osteoblastic cells. Zhu et al. (17) observed that BAIBA activates the reduced nicotinamide adenine dinucleotide phosphate (NADPH)/ROS signaling pathway in a dose- and time-dependent manner, thus promoting osteoblast differentiation. BAIBA intervention in MC3T3-E1 (mouse embryonic osteoblast precursor cells) cells resulted in a significant increase in ROS production, a significant acceleration of osteoblast proliferation and differentiation, and a significant increase in osteoblast transcriptional regulators, with mRNA expression increasing in a dose-dependent manner (17). The mRNA expression of osteoprotegerin (OPG) increased in a dose-dependent manner. In addition, NADPH oxidase (NOX) 4 expression was significantly upregulated in BAIBA-treated MC3T3-E1 cells, although it had no effect on the expression of NOX1 and NOX2 proteins in their family, suggesting that BIBA may induce ROS production in MC3T3-E1 cells through NOX4 to promote osteogenic differentiation. The Mas-related G protein-coupled receptor type D (MRGPRD) antagonist MU6840 blocked the effect of L-BAIBA on ROS induction (19), suggesting that L-BAIBA may have a direct effect on osteoblasts (18). The MRGPRD receptor was found to be significantly expressed in osteoblasts from juvenile mice, but was reduced in osteocytes from aged mice, which possibly indicated that the protective effect of BAIBA diminishes with age, not due to a diminished capacity of the muscle to produce BAIBA. The regulation of BAIBA function should be noted for changes in MRGPRD content in future studies. This could have important implications in the understanding of delay in skeletal aging due to exercise (18).

**Figure 3:**
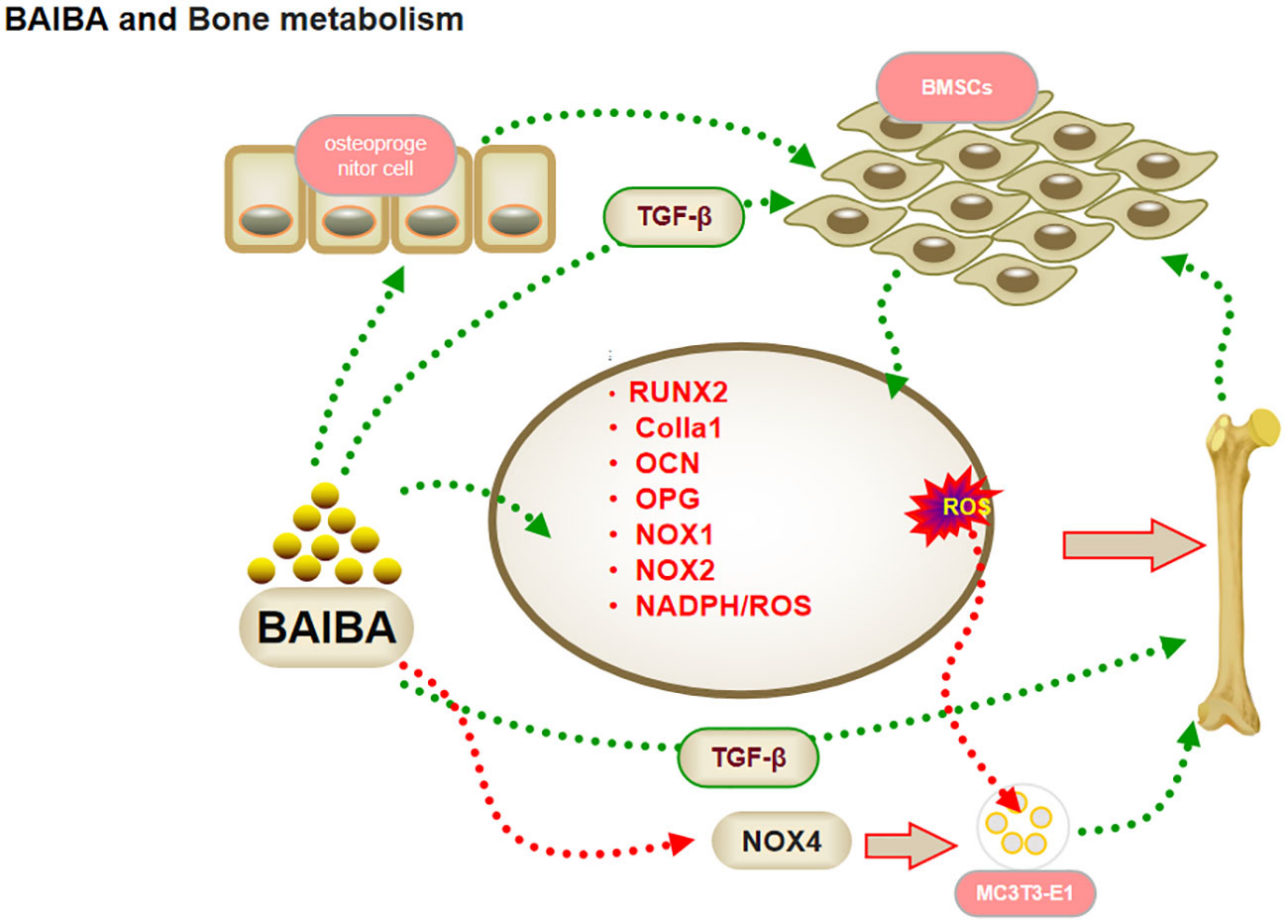
BAIBA and bone metabolism.

In summary, given the current new concept of muscle-bone crosstalk, the regulatory function of skeletal muscle signaling metabolites in bone may be more important than mechanical loading, with BAIBA promoting osteoblast proliferation and differentiation through NOX4-induced ROS production by osteoblast precursor cells, and TGF-b family members participating in cellular activity and metabolism during osteogenesis through the stimulation of BMSCs (51–53). Therefore, BAIBA may play an important regulatory role from bone progenitors to osteoblasts; however, further validation would be required to confirm this conclusion, since evidence is limited (**Figure 3**).

### 3.4 BAIBA improves inflammation

Inflammation is a defensive response of the body to stimuli; short-term inflammation helps to clear inflammatory substances, but chronic inflammation leads to structural and functional abnormalities in tissues and organs. Chronic inflammation of the hypothalamus, induced by saturated fatty acids, leads to proliferation and morphological changes in microglia, which are important regulators of energy metabolism (54–56). Park et al. (20) had reported that BAIBA treatment reverses palmitic acid-induced hypothalamic inflammation and microglia activation, and significantly reduces the expression of the inflammatory response factor cyclooxygenase 2 protein, hence suggesting that BAIBA may play a role in regulating chronic inflammation and protecting the body’s health. The energy regulators AMPK, protein kinase B (Akt), and insulin receptor substrate 1 (IRS-1) have been shown to regulate long-term inflammation through multiple pathways (57–59). Jung’s team explored the potential relationship between BAIBA and various other factors (8, 15, 16), and found that BAIBA significantly upregulates AMPK, AKT, and IRS-1 expression in 3T3T-L1 cells (15). Further studies showed that BAIBA significantly reverses the phosphorylation of pro-inflammatory factor IκBα, nuclear translocation of nuclear factor κB (NF-κB), serum tumor necrosis factor α (TNFα), and monocyte chemotactic protein-1 (MCP-1) in high-fat diet-fed mice and palmitate C2C12 skeletal muscle cells via the AMPK/PPARδ pathway. A follow-up study showed that BAIBA increased MCP-1 levels (8). The team subsequently showed that BAIBA could downregulate TNFα and MCP-1 expression in lipopolysaccharide-stimulated adipocytes and monocytes via the Akt/IRS-1 pathway and reduce lipopolysaccharide-induced monocyte adhesion to the endothelium (16).

Inflammation and impaired plasma lipid metabolism underlie the pathology of atherosclerosis (60). BAIBA inhibit TNFα-induced VCAM-1 and MCP-1 expression, and reduce atherosclerotic plaque area in ApoE-KO mice while having no effect on plasma lipids. This suggests that BAIBA could improve the inflammatory response and thus reduce VCAM-1 and MCP-1 expression, as well as the macrophage infiltration of plaque, ultimately inhibiting the development of atherosclerosis (35, 61).

**Figure 4:**
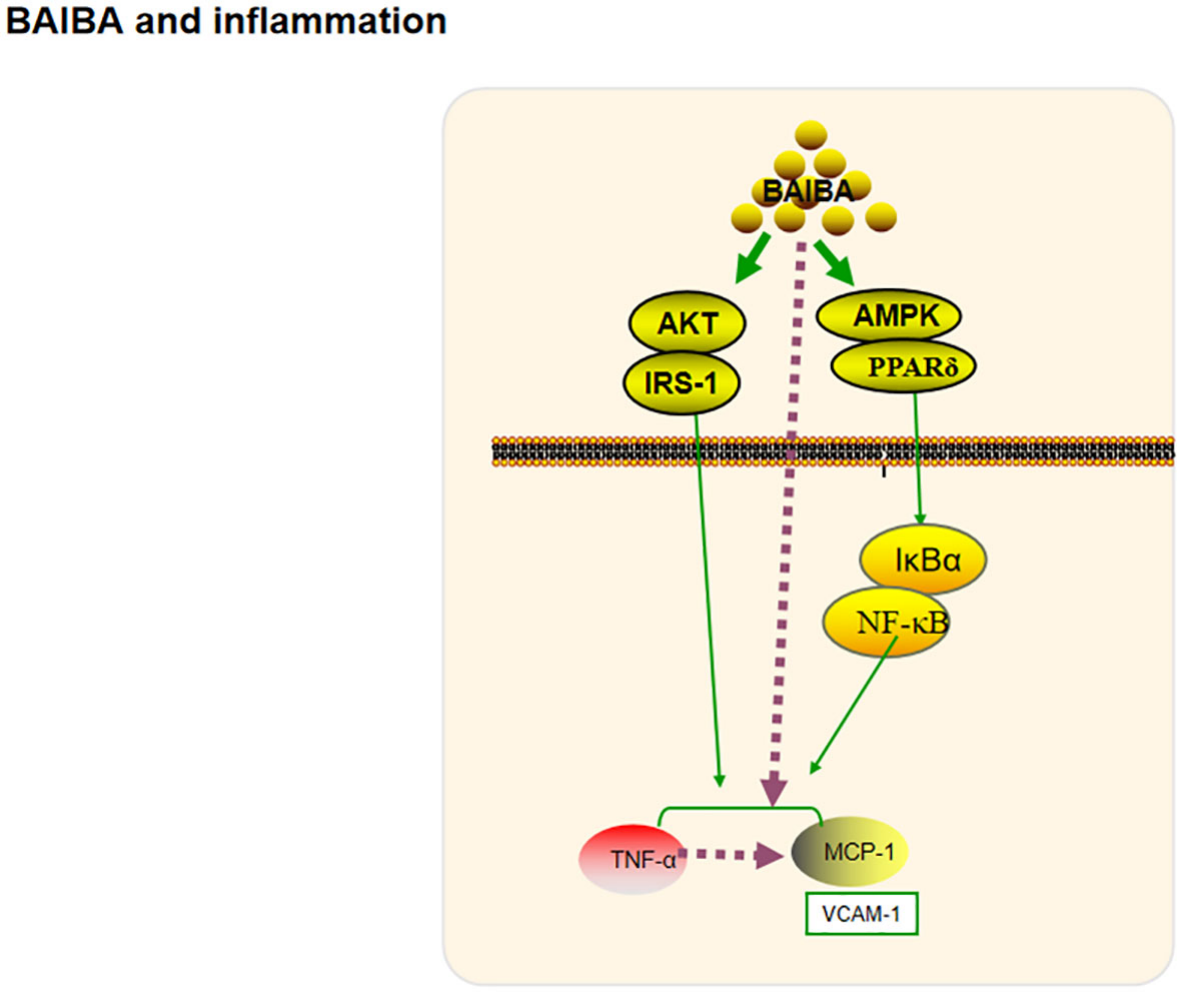
BAIBA and inflammation.

In summary, BAIBA might regulate the inflammatory response through AMPK and AKT pathways (**Figure 4**). Significant upregulation of the anti-inflammatory proteins, tyrosine protein kinase receptor and interleukin 2 receptor alpha chain protein, after high-dose BAIBA intervention was detected by serum proteomics, suggesting that BAIBA may directly affect the two proteins to mediate inflammation; notably, both were reported to be closely related to AKT expression (62, 63), although their relationship with AMPK expression has not been reported yet. Exploring the relationship between AMPK and the two proteins may be important to further reveal the anti-inflammatory effects of BAIBA in future.

### 3.5 BAIBA ameliorates oxidative stress

ROS and antioxidants are dynamically balanced in healthy organisms. When this balance is disturbed, increased ROS leads to impaired mitochondrial function and decreased nitric oxide synthase (NOS) expression, which in turn induces increased production of ROS in the electron transport chain, ultimately leading to oxidative stress (64, 65). One study reported that plasma BAIBA is downregulated in patients with novel coronavirus pneumonia and in health care workers severely exposed to SARS-CoV-2, and that citric acid, a marker of mitochondrial metabolic efficiency, is significantly downregulated (66). The significant correlation between BAIBA and mitochondria suggested that BAIBA may be closely related to oxidative stress (67). Several studies have shown that myocardial tissue lipid peroxidation, creatine kinase, malondialdehyde (MDA), and TNFα are downregulated, and hepatic MDA protein expression levels are decreased in diabetic mice after BAIBA injection (68), whereas the expression level of the antioxidant protein glutathione is upregulated (69). Wang et al. (70) observed that BAIBA pretreatment significantly improved the oxidative stress factor angiotensin II (Ang II) in NRK-49F (rat renal fibroblasts), possibly due to the decrease in ROS levels and increase in antioxidant factors induced by BAIBA pretreatment. Interestingly, AGXT2 showed the highest mRNA and protein expression levels in the kidneys (71). Therefore, L-BAIBA may play an important role in the amelioration of oxidative stress.

AMPK is an important factor for ameliorating oxidative stress, promoting NO synthesis, and improving mitochondrial function. 2022 research showed that BAIBA upregulated AMPK phosphorylation levels, NO, L-arginine, malate (substrates for NO synthesis), NOS activity, and ADP/ATP ratio levels in the renal medulla of hypertensive rats (72, 73). BAIBA may improve oxidative stress by phosphorylating AMPK and increasing NO levels (72, 73). Further studies showed that BAIBA improved mitochondrial morphology, downregulated the expression of optic atrophy protein 1 and mitochondrial dynamics-related protein, and downregulated ATP production in the left ventricle of rats with heart failure through AMPK/miR-208b, thereby improving mitochondrial quality (74); this suggested that AMPK may be central to BAIBA-mediated mitochondrial function and amelioration of oxidative stress.

Sawada et al. (65) had reported that BAIBA can significantly reduce oxidative stress in vascular endothelial cells by regulating the mitochondrial function-related factor PGC-1β-estrogen-related receptor (ERRα)/PPAR-δ/PPAR-γ pathway (65). BAIBA intervention significantly upregulated the oxidative stress response of TNF cells. Moreover, BAIBA intervention significantly upregulated the TNF-α-mediated antioxidant factors CAT, SOD, thioredoxin, and γ-glutamylcysteine ligase in aortic endothelial cells and human umbilical vein endothelial cells, mitochondrial biogenesis-related molecular nuclear respiration Factor 1 and mitochondrial transcription factor mRNA levels. Notably, BAIBA treatment was observed to increase the expression of PGC-1β-downstream factors PPAR-δ, PPAR-γ, and ERRα before activating antioxidants and mitochondrial effects, hence suggesting that BAIBA takes longer to exert its biological effects by ameliorating oxidative stress and could involve multiple transcriptional steps (65). This study differed from previous reports, since significant changes in AMPK, Akt, and NOS phosphorylation were not observed after long- and short-term BAIBA treatment, which could be due to the different cell types used, suggesting that future studies on the mechanisms of BAIBA-mediated oxidative stress in different cells.

**Figure 5:**
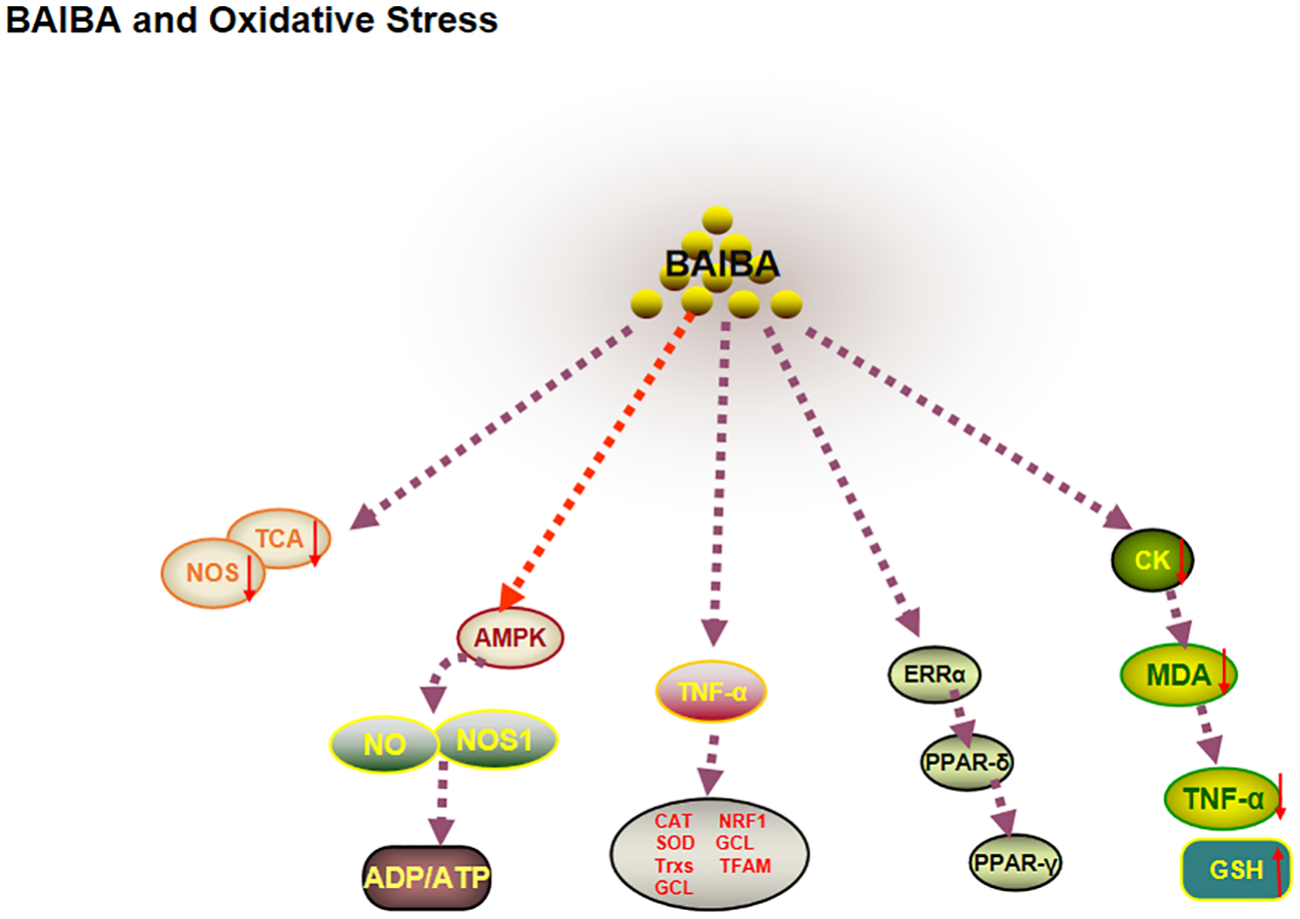
BAIBA and oxidative stress.

In summary, BAIBA affects mitochondrial genesis and ROS synthesis capacity via AMPK, thereby regulating oxidative stress (**Figure 5**).

## 4 Exercise regulates BAIBA synthesis and metabolism

BAIBA acts as a signaling metabolite and an antioxidant that is regulated by exercise, as validated at multiple levels (animal, cellular, protein, and genetic) (73–75).

Serum BAIBA levels are not significantly altered in adult male participants either immediately after acute aerobic exercise (70% of maximal oxygen uptake), or after 1 h, or even 4 h (75). Interestingly, serum D-BAIBA and L-BAIBA levels increased by 13% and 20%, respectively, after 1 h of acute aerobic exercise (40% maximal power) in 15 subjects (12 female and 3 male) with a polymorphism (rs37369) of a different (AGXT) genotype, which could be due to the fact that AGXT upregulated and catabolized D-BAIBA similarly during acute exercise (76), and L-BAIBA production increased, possibly due to the oxidation of the upstream species L-valine (18). This suggested that future assessment of exercise-mediated changes related to BAIBA would require special attention to isomeric differences and changes in AGXT (75, 77).

With regard to long-term exercise, in the follow-up of patients undergoing renal dialysis, plasma BAIBA levels were found to be significantly lower in sedentary patients than in those who remained physically active over long periods of time; however, there was no significant difference in the data across subjects with different levels of exercise over a long term. Unfortunately, owing to its cross-sectional nature, a causal relationship between plasma BAIBA levels and physical activity could not be established in the study (76). Morales et al. (75) reported a significant increase in serum BAIBA levels after 20 weeks of aerobic training. A study including both normal and obese adolescents showed that serum BAIBA levels increased after prolonged exercise (aerobic or resistance) regardless of obesity (78). This was confirmed by the results of an animal model, in which four weeks of hypoxic training increased BAIBA levels in the gastrocnemius and blood of obese rats (79). Roberts et al. (4) found serum BAIBA levels to be significantly higher in mice subjected to 3 weeks of free wheel exercise than in quiet control mice, and the BAIBA levels in gastrocnemius and quadriceps to be increased 5.2-fold and 2.2-fold, respectively. This suggested that exercise significantly up-regulates BAIBA levels in muscle and serum. Differences in the modulation of BAIBA between different forms of exercise would be worth noting (80). Research observed that high-intensity interval training (HIIT) and moderate-intensity continuous training (MICT) significantly upregulated the expression of HADH, ACADS, and HADHA, key genes involved in BAIBA biosynthesis, in APOE-KO mice fed a high-fat diet. The mRNA expression of HADH, ACADS, and HADHA, key genes involved in BAIBA biosynthesis, was significantly upregulated in the HIIT group. Interestingly, after two weeks of single-leg immobilization, the femoral artery and femoral plasma concentrations in each leg were significantly higher in healthy male subjects at rest and during knee extension exercise in both legs. Venous plasma BAIBA concentrations were increased, but static-exercise BAIBA concentrations were decreased after leg immobilization (39). This pulsatile pattern of release and uptake may be a result of BAIBA uptake by skeletal muscle and may also be related to the metabolism of thymidine as a result of exercise.

In summary, although the modulatory effects of acute exercise on BAIBA levels have been studied relatively less, different forms of long-term exercise significantly increased BAIBA levels in serum and skeletal muscle, and HIIT has a more significant upregulation effect on serum and skeletal muscle BAIBA However, comparisons between different forms of exercise are currently scarce; therefore, the effect of factors, such as intensity, duration, and rest, on BAIBA levels would need to be explored further, and the question of whether exercise regulates synthesis and breakdown of BAIBA or uptake of BAIBA by skeletal muscle will be central to uncovering the relationship between exercise and BAIBA.

Little is known about the mechanisms by which BAIBA mediates exercise to regulate metabolism. 2022 research showed that BAIBA levels are reduced in hypoxic environments, whereas BAIBA expression is increased in blood and gastrocnemius muscle of mice after hypoxic exercise and was positively correlated with inguinal fat PPARα expression levels (81). Notably, both the hypoxic environment and hypoxic exercise upregulated PPARα mRNA expression in adipose tissue (81). Whether hypoxic exercise, as an effective form of fat loss, can promote fat metabolism by regulating adipose PPARα levels through BAIBA, and the mechanism of hypoxic environment being an external stimulus in BAIBA production during exercise would be important directions for future research towards revealing the mechanism of hypoxic exercise for weight loss.

To date, studies have shown that moderate exercise produces ROS, which promotes AMPK activation. Intense exercise, on the other hand, impairs mitochondrial function and inhibits AMPK activity and expression. Antioxidant supplementation may ameliorate the damage caused by excessive ROS production from intense exercise, but it counteracts the beneficial effects of moderate exercise at the same time, resulting in two incompatible interventions (82). A recent study comparing exercise and antioxidant interventions in T2DM rats found that moderate exercise can promote redox reactions in the liver by increasing antioxidant capacity. In contrast, antioxidant interventions impair redox homeostasis by inhibiting oxidative stress (82). It is interesting to note that BAIBA acts as a signaling metabolite and antioxidant, regulated by exercise; this has been validated at multiple levels (animal, cellular, protein, and genetic) (72–74). The validation of AMPK as a downstream target of BAIBA suggests that BAIBA supplementation may generate moderate ROS and serve as a suitable intervention to promote redox homeostasis while avoiding the damage caused by intense exercise (82).

## 5 Prospects of BAIBA being used as an exercise pill?

Studies have shown that BAIBA plays an important role in health, not only by effectively improving glucose and lipid metabolism, but also by improving bone homeostasis and downregulating inflammatory responses and oxidative stress. A number of these effects appear to parallel benefits obtained via exercise-generated BAIBA, suggesting that BAIBA could potentially be applied as a drug or exercise mimetic.

In clinical applications, it is important to achieve drug efficacy without toxic side effects. A toxicological study showed that L-BAIBA can be delivered without adverse effects at 900 mg/kg/day for 90 days, which is the highest level tested (83). In addition, in human studies, approximately 2–20% Caucasians and 50% Asians were found to be deficient in AGXT2 (84, 85), with high urinary D-BAIBA levels. Kittel et al. (86) detected three-fold higher plasma BAIBA concentrations in a small number of subjects with AGXT2 gene deficit; however, none of the studies reported any adverse effect of increased endogenous BAIBA, suggesting that endogenous and exogenous factors resulting in high plasma BAIBA levels do not have any pathological effect (87). Notably, in pathological situations with substantial DNA degradation, such as malignancy (84, 88), radiation (89), and short-term starvation (90), the levels of BAIBA in urine are elevated. However, the current study was unable to define a causal relationship between BAIBA and these pathological conditions, and given that BAIBA inhibits oxidative stress in cancer cells (13), the feedback elevation of BAIBA was speculated to be a protective strategy of the body itself, thereby providing a theoretical basis for the clinical application of BAIBA as a new drug.

## 6 Applicability of BAIBA as a useful biomarker?

Although BAIBA can be detected in both blood and urine, the time course in the two matrices may not be the same. After an intervention with the DNA-based drug BC007, plasma BAIBA concentrations were found to increase after 3 min, reached a maximum after 60 min, and declined to baseline levels (87). The same intervention led to a rise in urinary BAIBA levels within a maximum of 2−4 h and a decline to pre-intervention levels over 8–12 h. BAIBA was considered to be metabolized rapidly in plasma, with urine representing a better matrix for testing, since sampling is non-invasive and the time profile is more favorable for testing.

Wang et al. reported that D-BAIBA is predominantly synthesized in humans while L-BAIBA is predominantly synthesized in mice (45); this was consistent with the recent finding that human D-BAIBA plasma concentrations are 67-fold higher than those of L-BAIBA (77). Most studies have shown that D-BAIBA is the major enantiomer of BAIBA in human urine (4, 91). However, in the mammalian kidney, AGXT2 levels are high (71), and D-BAIBA is considered the preferred substrate (23); therefore, D-BAIBA would be difficult to detect in urine (92). Since the lowest levels of urinary BAIBA were not observed in the 24-h observation time frame, D-MMS could have been further metabolized. Therefore, detection of the catabolite D-MMS may be the next step in determining whether D-BAIBA can be used as a urinary disease biomarker.

Biomarkers are characterized by their specificity, sensitivity, stability, non-invasiveness, and reproducibility. BAIBA is considered a biomarker for osteoporosis (45), and recent studies have shown it to possibly be a biological marker for the coronavirus infection as well (67). While two histological screens showed that BAIBA specificity and reproducibility met requirements (45, 68), and non-invasive testing in urine is attractive, there are several issues regarding the detection of enantiomers and metabolic degradation of BAIBA, owing to which stability and sensitivity of assays for BAIBA as a biological marker would need to be explored further.

## 7 Concluding remarks and future perspectives

BAIBA is a signaling metabolite that maintains lipid homeostasis by promoting white fat browning and increasing FFA oxidation, lowers blood glucose (possibly through an insulin-independent pathway), promotes osteogenic differentiation, and reduces inflammation and oxidative stress. Exercise promotes BAIBA production in skeletal muscle and inhibits its uptake. BAIBA has effects in a variety of diseases and has become a biomarker for osteoporosis and the coronavirus infection. Exogenous BAIBA can effectively perform biological functions without causing any side effects. Thus, it may have a promising future as a drug and biomarker.

However, there are many questions still that need to be addressed in future. 1. Since most existing epidemiological and experimental studies do not distinguish between D-BAIBA and L-BAIBA, there is a need to further define the biological functions of each subtype. 2. Since osteoclasts form an important factor in bone metabolism, it would be important to explore whether BAIBA can directly mediate the number or function of osteoclasts, and what would be the mechanism involved. 3. There are only few studies that have compared the regulation of BAIBA levels by different exercise modalities, and investigated whether exercise regulates health via BAIBA; hence, the relationship between exercise and BAIBA synthesis and secretion should be explored in detail in future using different times, intensities, and exercise frequencies.

## Author contributions

XY, YY is responsible for topic selection, frame design and writing. TL, ML, TY, GH and GW were responsible for document retrieval and chart design. BC conducted literature research and final revisions. All authors contributed to the article and approved the submitted version.
